# Cardiovascular therapy use, modification, and in-hospital death in patients with COVID-19: A cohort study

**DOI:** 10.1371/journal.pone.0277653

**Published:** 2022-11-23

**Authors:** Cédric Follonier, Elena Tessitore, Sandra Handgraaf, David Carballo, Maëlle Achard, Antoinette Pechère-Bertschi, François Mach, François R. Herrmann, François R. Girardin

**Affiliations:** 1 Division of Cardiology, Department of Medicine, Geneva University Hospitals, Geneva, Switzerland; 2 Faculty of Medicine, University of Geneva, Geneva, Switzerland; 3 Division of Nephrology and Hypertension, Department of Medicine, Geneva University Hospitals, Geneva, Switzerland; 4 Division of Geriatrics, Department of Rehabilitation and Geriatrics, Geneva University Hospitals, Geneva, Switzerland; 5 Division of Clinical Pharmacology, Department of Laboratory Medicine and Pathology, Lausanne University Hospital, Faculty of Medicine, University of Lausanne, Lausanne, Switzerland; 6 Division of Clinical Pharmacology and Toxicology, Department of Anaesthesiology, Clinical Pharmacology, Intensive Care and Emergency Medicine, Geneva University Hospitals, Geneva, Switzerland; Madras Medical College, INDIA

## Abstract

**Aims:**

To assess the associations of exposure and modifications in exposure (i.e., discontinuation on admission, initiation during hospitalization) to eight common cardiovascular therapies with the risk of in-hospital death among inpatients with coronavirus disease 2019 (COVID-19).

**Methods:**

In this observational study including 838 hospitalized unvaccinated adult patients with confirmed COVID-19, the use of cardiovascular therapies was assessed using logistic regression models adjusted for potential confounders.

**Results:**

No cardiovascular therapy used before hospitalization was associated with an increased risk of in-hospital death. During hospitalization, the use of diuretics (aOR 2.59 [1.68–3.98]) was associated with an increase, and the use of agents acting on the renin-angiotensin system (aOR 0.39 [0.23–0.64]) and lipid-lowering agents (aOR 0.41 [0.24–0.68]) was associated with a reduction in the odds of in-hospital death. Exposure modifications associated with decreased survival were the discontinuation of an agent acting on the renin-angiotensin system (aOR 4.42 [2.08–9.37]), a β-blocker (aOR 5.44 [1.16–25.46]), a lipid-modifying agent (aOR 3.26 [1.42–7.50]) or an anticoagulant (aOR 5.85 [1.25–27.27]), as well as the initiation of a diuretic (aOR 5.19 [2.98–9.03]) or an antiarrhythmic (aOR 6.62 [2.07–21.15]). Exposure modification associated with improved survival was the initiation of an agent acting on the renin-angiotensin system (aOR 0.17 [0.03–0.82]).

**Conclusion:**

In hospitalized and unvaccinated patients with COVID-19, there was no detrimental association of the prehospital use of any regular cardiovascular medication with in-hospital death, and these therapies should be continued as recommended.

## Introduction

Severe acute respiratory syndrome coronavirus 2 (SARS-CoV-2) infection substantially impacts the heart and the cardiovascular system with complex inflammatory processes and circulation disorders. COVID-19-related cardiovascular manifestations include a wide range of diseases, such as myocardial injury, thromboembolic events, arrhythmias, and acute heart failure [1–4]. Major cardiovascular adverse events were strongly associated with COVID-19 severity and mortality [5, 6].

The binding of the virus spike protein to angiotensin-converting enzyme 2 (ACE2) is a key component of the renin-angiotensin system [7]. Typical patient characteristics and medications appear to be determinant because ACE2 is widely expressed on the surface of pulmonary alveolar cells and on the endothelial cells of high-perfusion organs, which could affect the clinical course by SARS-CoV-2 infection and inflammatory processes [8, 9]. Angiotensin II is significantly higher in individuals with SARS-CoV-2 infection: higher plasma concentration was associated with viral load and disease severity [10, 11]. The role of ACE2 has led to concern about antihypertensive drugs, including renin-angiotensin system inhibitors (RASis) [12, 13].

Although most hospitalized patients with COVID-19 are exposed to cardiovascular medications, evidence of sequential drug exposure on disease severity, mortality, and long-term outcomes is still unclear. We hypothesized that cardiovascular medication before and during hospitalization is safe and not associated with increased mortality in hospitalized patients with COVID-19.

## Methods

### Study design and population

Data were derived from the Geneva COVID-19 Cardiovascular Study (GCCS, ClinicalTrials.gov Identifier: NCT04384029) to assess the associations of cardiovascular therapies with all-cause mortality in patients hospitalized for COVID-19. The GCCS included unvaccinated adult patients hospitalized for COVID-19 with confirmed SARS-CoV-2 infection at the University Hospitals of Geneva, Switzerland, between February 26, 2020, and April 26, 2020. All patients discharged or deceased by June 5, 2020, were included. The inclusion period covered the first wave of the pandemic in the Geneva region. The inclusion criteria were age ≥18 years, a positive test for SARS-CoV-2 (i.e., a nasopharyngeal swab, broncho-alveolar lavage, or blood test for antibodies), and hospitalization for COVID-19-related symptoms. Patients who previously refused to provide general research consent, those included in an interventional study, or those who remained hospitalized as of June 5, 2020, were not included in the GCCS. The Geneva University Hospitals were the only referral center to admit patients with COVID-19 requiring hospitalization for the entire Geneva area (population of 500,000 inhabitants). The study protocol was evaluated and accepted by the Geneva Research Ethics Committee (protocol 2020–00610) with a waiver of *ad hoc* informed consent.

### Data sources and collection

Demographics, medical history, coexisting conditions, cardiovascular risk factors, laboratory parameters, and at-home and hospitalization medications were collected and analyzed by a trained team of medical staff in the Department of Cardiology based on electronic medical records. Validated automated feeds identified patients from the electronic records, and a trained team manually entered their data into REDCap®, a secure web platform for building and managing online databases [14]. The research was performed based on the Reporting of Observational Studies in Epidemiology (STROBE) guidelines [15].

### Outcome and predictors

The main outcome of this study was all-cause in-hospital death. Predictors included the exposure status to common cardiovascular drugs, which were grouped into eight categories according to the Anatomical Therapeutic Chemical (ATC) Index 2021 established by the WHO Collaborating Centre for Drug Statistics Methodology [16]: agents acting on the renin-angiotensin system, β-blocking agents, calcium channel blockers, diuretics, lipid-modifying agents, antithrombotics, and antiarrhythmics (classes II and III). Antithrombotics were further divided into antiplatelets and anticoagulants (direct oral anticoagulants, vitamin K antagonists, and heparins). If several substances were administered in combination in a single preparation, each was recorded individually.

Prehospital drug exposure was defined as the active prescription of the drug at the time of admission. Modifications in drug exposure were defined as follows: in patients with prehospital exposure to the drug, discontinued if stopped on hospital admission; in patients with no prehospital exposure to the drug, initiated if started during hospitalization. In-hospital use was defined as the use of the drug at any time during hospitalization for COVID-19.

Coexisting conditions included cardiovascular risk factors, cardiovascular disease, diabetes (type I or II), respiratory disease, moderate or severe chronic kidney disease, chronic liver disease, and active malignancy. Cardiovascular risk factors were defined as hypertension, obesity, dyslipidemia, active smoking, physical inactivity, stress, and family history of cardiovascular disease. Cardiovascular disease included a history of acute coronary syndrome (ST-segment elevation myocardial infarction (STEMI) and non-STEMI (NSTEMI), unstable angina (UA)), ischaemic or hemorrhagic stroke, major arrhythmias (atrial fibrillation, atrial flutter, ventricular tachycardia, or ventricular fibrillation), and heart failure. Respiratory diseases were defined as chronic obstructive pulmonary disease, asthma, and sleep apnoea. Cardiovascular complications occurring during hospitalization included acute coronary syndrome (either STEMI, NSTEMI, or UA), major arrhythmia (atrial fibrillation, atrial flutter, ventricular tachycardia, ventricular fibrillation), acute heart failure, ischaemic or hemorrhagic stroke, and acute venous thromboembolism (deep vein thrombosis, pulmonary embolism). The individual diagnoses of cardiovascular complications, as well as the definition of COVID-19 severity, are provided in **S1 File**.

### Statistical analysis

Quantitative variables are presented as the means with standard deviations or the medians with interquartile ranges, and qualitative variables are shown using frequency tables and percentages. P values are reported using Student’s t test-test, Fisher’s exact test, or the Wilcoxon-Mann-Whitney test when appropriate and without adjustments for multiple testing. The statistical significance threshold was set at p ≤ 0.05.

The drug sequence and dose modification prior to and during hospitalization were reported and analyzed for each cardiovascular therapy. Patients were divided into two groups: one including patients with prehospital exposure and one including patients without prehospital exposure to the drug of interest. Patients with prehospital exposure were further divided into two groups: those who continued the therapy and those who discontinued it during hospitalization. Patients without prehospital exposure were further divided into two groups: those who initiated the therapy during hospitalization and those who did not. When the use of a drug or a coexisting condition was not documented in the electronic medical record, the patient was considered unexposed to it.

The associations of prehospital and in-hospital exposure and modifications in exposure (i.e., discontinuation, initiation) to cardiovascular therapies with the risk of in-hospital death were analyzed using multiple logistic regression models adjusted for age (quartiles) and the following confounders, all treated as binary variables: male sex, cardiovascular risk factor, history of cardiovascular disease, diabetes, renal disease, respiratory disease, liver disease, and malignancy. This set of confounders is the standard in similar papers dealing with the topic [17–19].

Regarding power calculation, with a number of events per variable in multiple logistic regression analysis of 10 or more, there should be no bias in the results [20, 21]. Thus with 152 non-survivors and 686 survivors, up to 15 variables can be included in each multiple logistic model including the entire cohort [22].

Statistical analyses were performed using Stata software version 17 (Stata Corp., College Station, TX, USA).

## Results

**Fig 1:**
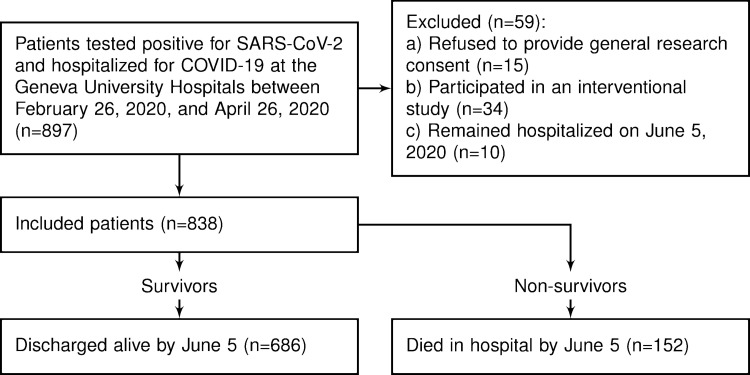
Study flowchart.

During the study period, 838 patients had been hospitalized for a confirmed SARS-CoV-2 infection and were discharged alive or died during hospitalization at the Geneva University Hospitals (**Fig 1**).

Of these, 453 (54.1%) were male, 102 (12.2%) were admitted to the intensive care unit, and 152 (18.1%) were deceased during the hospital stay. The mean age was 66.51 ± 17.6 years. Deceased patients were more likely to suffer from coexisting conditions and to develop cardiovascular complications during hospitalization. Patient characteristics are described in **Table 1.**

**Table 1:** Demographic and clinical characteristics of patients hospitalized with COVID-19.

	Total	Survivors	Nonsurvivors	P value
Total	838 (100.0)	686 (81.9)	152 (18.1)	
**Patient characteristics**				
Age (years)	66.5 ± 17.6	63.2 ± 17.1	81.4 ± 10.6	<0.001
Male sex	453 (54.1)	357 (52.0)	96 (63.2)	0.013
**Cardiovascular risk factors**				
Cardiovascular risk factors	605 (72.2)	485 (70.7)	120 (78.9)	0.040
Hypertension	392 (46.8)	297 (43.3)	95 (62.5)	<0.001
Obesity	286 (34.1)	246 (35.9)	40 (26.3)	0.025
Active smoking	37 (4.4)	31 (4.5)	6 (3.9)	0.756
Dyslipidaemia	209 (24.9)	162 (23.6)	47 (30.9)	0.060
Physical inactivity	22 (2.6)	11 (1.6)	11 (7.2)	0.000
**Coexisting conditions**				
Cardiovascular disease	238 (28.4)	150 (21.9)	88 (57.9)	<0.001
Acute coronary syndrome	95 (11.3)	56 (8.2)	39 (25.7)	<0.001
Stroke	78 (9.3)	55 (8.0)	23 (15.1)	0.006
Arrhythmia	104 (12.4)	65 (9.5)	39 (25.7)	<0.001
Heart failure	60 (7.2)	27 (3.9)	33 (21.7)	<0.001
Diabetes (types I and II)	169 (20.2)	122 (17.8)	47 (30.9)	<0.001
Respiratory disease	164 (19.6)	129 (18.8)	35 (23.0)	0.235
Chronic kidney disease	96 (11.5)	53 (7.7)	43 (28.3)	<0.001
Liver disease	34 (4.1)	26 (3.8)	8 (5.3)	0.405
Active malignancy	43 (5.1)	25 (3.6)	18 (11.8)	<0.001
**Hospitalization**				
Hospitalization length (days)	13.1 ± 11.1	13.3 ± 11.6	12.4 ± 8.5	0.373
ICU stay	102 (12.2)	71 (10.3)	31 (20.4)	0.001
**Outcomes**				
Cardiovascular events	160 (19.1)	87 (12.7)	73 (48.0)	<0.001
Acute coronary syndrome	18 (2.1)	11 (1.6)	7 (4.6)	0.021
Arrhythmia	45 (5.4)	28 (4.1)	17 (11.2)	<0.001
Heart failure	89 (10.6)	41 (6.0)	48 (31.6)	<0.001
Stroke	10 (1.2)	6 (0.9)	4 (2.6)	0.089
Acute venous thromboembolism	27 (3.2)	16 (2.3)	11 (7.2)	0.002
**COVID-19 WHO severity index**				
Mild or moderate	86 (10.3)	83 (12.1)	3 (2.0)	
Severe	605 (72.2)	517 (75.4)	88 (57.9)	
Critical	147 (17.5)	86 (12.5)	61 (40.1)	<0.001

Demographic and clinical characteristics of patients hospitalized with COVID-19. Continuous variables are reported as the mean ± standard deviation (SD), and categorical variables are reported as N (%).

### Cardiovascular drug use

**Fig 2:**
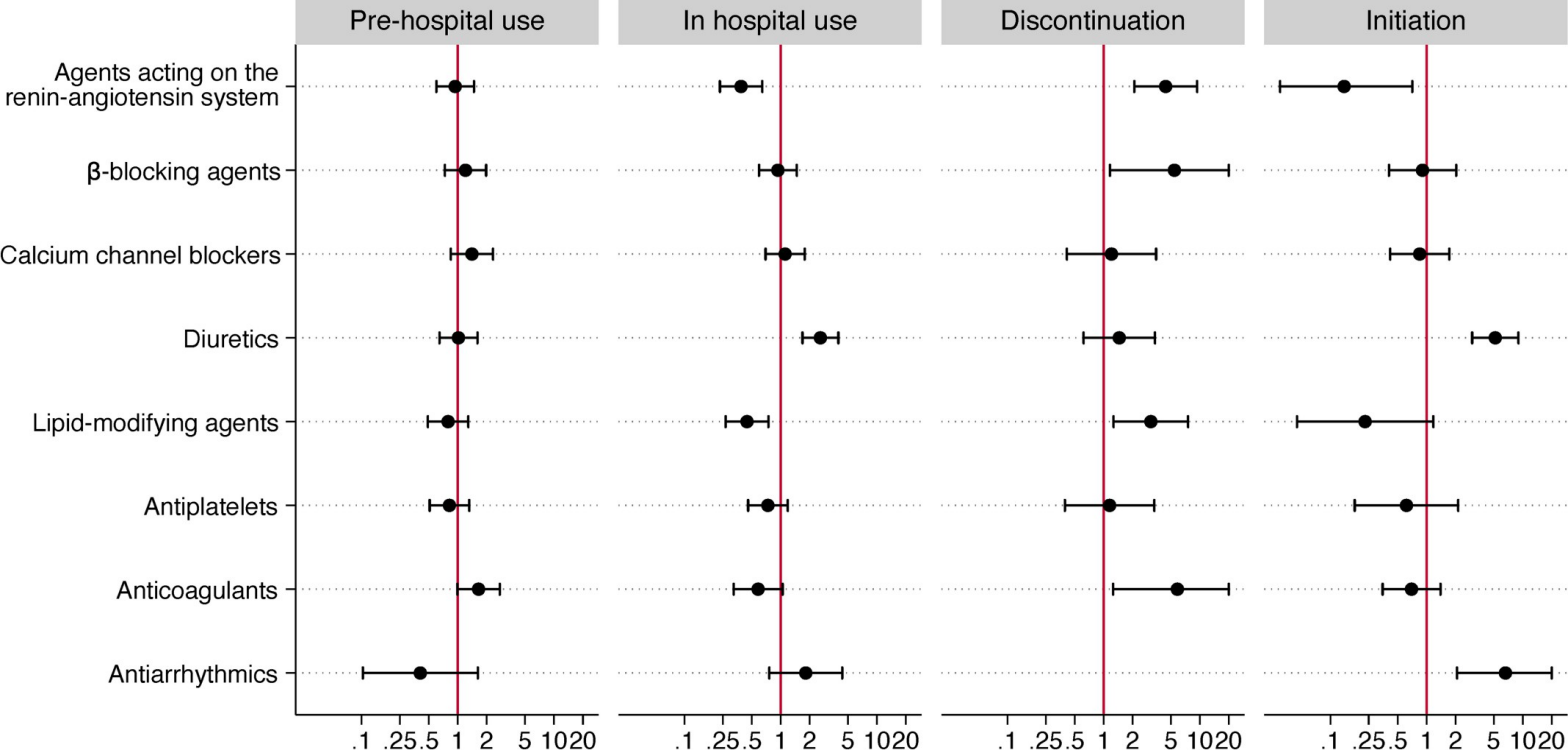
Cardiovascular drug use and modification analysis. Odds ratios with 95% confidence intervals for in-hospital death for prehospital use (vs. no prehospital use), in-hospital use (vs. no in-hospital use), discontinuation (vs. continuation), and initiation (vs. absence). Odds ratios were adjusted for age, sex, cardiovascular risk factors, and comorbidities.

Cardiovascular medications were prescribed to 468 (55.8%) patients before hospitalization, and 779 (92.5%) had at least one of the cardiovascular drug prescriptions modified within the first three days following hospital admission. After adjusting for potential confounding factors, except anticoagulants (aOR 1.66 (1.00–2.77), p = 0.049), no cardiovascular therapies administered before hospitalization were significantly associated with in-hospital mortality (**Table 2 and Fig 2**).

**Table 2:** Association of prehospital cardiovascular therapy exposure with the risk of in-hospital death in patients with COVID-19.

*Prehospital use (*vs. *no prehospital use)*	Prehospital use	No prehospital use	Crude OR		Adjusted OR †	
	*N (%)*	*N (%)*	*(95% CI)*	*P value*	*(95% CI)*	*P value*
Agents acting on the renin-angiotensin system	61/268 (22.8)	91/570 (16.0)	1.55 (1.08–2.23)	0.018	0.94 (0.60–1.47)	0.784
ß-blocking agents	55/156 (35.3)	97/682 (14.2)	3.28 (2.22–4.86)	<0.001	1.20 (0.73–1.97)	0.463
Calcium channel blockers	40/123 (32.5)	112/715 (15.7)	2.59 (1.69–3.98)	<0.001	1.40 (0.85–2.32)	0.190
Diuretics	60/200 (30)	92/638 (14.4)	2.54 (1.75–3.70)	<0.001	1.02 (0.64–1.60)	0.947
Lipid-modifying agents	53/202 (26.2)	99/636 (15.6)	1.93 (1.32–2.82)	0.001	0.79 (0.49–1.29)	0.346
Antiplatelets	48/180 (26.7)	104/658 (15.8)	1.94 (1.31–2.86)	0.001	0.82 (0.51–1.31)	0.406
Anticoagulants	57/133 (42.9)	95/705 (13.5)	4.82 (3.21–7.23)	<0.001	1.64 (0.99–2.73)	0.055
Antiarrhythmics	3/16 (18.8)	149/822 (18.1)	1.04 (0.29–3.70)	0.949	0.41 (0.10–1.61)	0.201

Association of prehospital cardiovascular therapy exposure with the risk of in-hospital death. Patient counts are reported as N (%), and odd ratios with their 95% confidence intervals are presented on a logarithmic scale.

†The models were adjusted for age, sex, cardiovascular risk factors, and coexisting conditions as described in the Methods.

During hospitalization, the use of diuretics (aOR 2.59 [1.68–3.98], p<0.001) was associated with an increase in the odds of in-hospital death, whereas the use of agents acting on the renin-angiotensin system (aOR 0.39 [0.23–0.64], p<0.001) and lipid-lowering agents (aOR 0.41 [0.24–0.68], p = 0.001) were associated with a reduction in the odds of in-hospital death. (**Table 3**).

**Table 3:** Association of in-hospital use of cardiovascular therapies with the risk of in-hospital death in patients with COVID-19 with prehospital exposure to the treatment of interest.

*In-hospital use (*vs. *no in-hospital use)*	In-hospital use	No in-hospital use	Crude OR		Adjusted OR †	
	*N (%)*	*N (%)*	*(95% CI)*	*P value*	*(95% CI)*	*P value*
Agents acting on the renin-angiotensin system	33/210 (15.7)	119/628 (18.9)	0.80 (0.52–1.22)	0.293	0.39 (0.23–0.64)	<0.001
ß-blocking agents	55/213 (25.8)	97/625 (15.5)	1.89 (1.30–2.76)	0.001	0.93 (0.59–1.47)	0.771
Calcium channel blockers	41/177 (23.2)	111/661 (16.8)	1.49 (1.00–2.24)	0.052	1.11 (0.70–1.78)	0.660
Diuretics	104/305 (34.1)	48/533 (9.0)	5.23 (3.58–7.64)	<0.001	2.59 (1.68–3.98)	<0.001
Lipid-modifying agents	35/169 (20.7)	117/669 (17.5)	1.23 (0.81–1.88)	0.332	0.41 (0.24–0.68)	0.001
Antiplatelets	45/180 (25.0)	107/657 (16.3)	1.70 (1.15–2.53)	0.009	0.74 (0.46–1.18)	0.207
Anticoagulants	127/726 (17.5)	25/112 (22.3)	0.74 (0.45–1.20)	0.218	0.58 (0.32–1.05)	0.070
Antiarrhythmics	11/30 (36.7)	141/808 (17.5)	2.74 (1.28–5.88)	0.010	1.82 (0.76–4.36)	0.178

Association of in-hospital use of cardiovascular therapies with the risk of in-hospital death in patients with prehospital exposure to the treatment of interest. Patient counts are reported as N (%), and odd ratios with their 95% confidence intervals are presented on a logarithmic scale.

†The models were adjusted for age, sex, cardiovascular risk factors, and coexisting conditions.

### Cardiovascular drug exposure modifications

In patients with prehospital exposure, the discontinuation on admission of an agent acting on the renin-angiotensin system (aOR 4.42 [2.08–9.37], p<0.001), a β-blocker (aOR 5.44 [1.16–25.46], p = 0.031), a lipid-modifying agent (aOR 3.26 [1.42–7.50], p = 0.005) and an anticoagulant (aOR 5.85 [1.25–27.27], p = 0.025) was associated with a significant increase in the odds of in-hospital death. No cardiovascular drug discontinuation was associated with improved survival (**Table 4 and Fig 2**).

**Table 4:** Association of discontinuation of cardiovascular therapies with the risk of in-hospital death in patients with COVID-19, with prehospital exposure to the treatment of interest.

*Discontinuation (*vs. *continuation)*	Discontinuation	Continuation	Crude OR		Adjusted OR †	
	*N (%)*	*N (%)*	*(95% CI)*	*P value*	*(95% CI)*	*P value*
Agents acting on the renin-angiotensin system	30/86 (34.9)	31/182 (17)	2.61 (1.45–4.70)	0.001	4.42 (2.08–9.37)	<0.001
ß-blocking agents	9/14 (64.3)	46/142 (32.4)	3.76 (1.19–11.84)	0.024	5.44 (1.16–25.46)	0.031
Calcium channel blockers	12/30 (40.0)	28/93 (30.1)	1.55 (0.66–3.64)	0.316	1.21 (0.42–3.51)	0.730
Diuretics	13/47 (27.7)	47/153 (30.7)	0.86 (0.42–1.78)	0.689	1.45 (0.62–3.42)	0.390
Lipid-modifying agents	20/54 (37)	33/148 (22.3)	2.05 (1.04–4.02)	0.037	3.26 (1.42–7.50)	0.005
Antiplatelets	7/26 (26.9)	41/154 (26.6)	1.02 (0.40–2.59)	0.975	1.15 (0.40–3.37)	0.794
Anticoagulants	10/13 (76.9)	47/120 (39.2)	5.18 (1.35–19.80)	0.016	5.85 (1.25–27.27)	0.025
Antiarrhythmics	1/3 (33.3)	2/13 (15.4)	2.75 (0.16–46.79)	0.484	.	.

Association of discontinuation of cardiovascular therapies with the risk of in-hospital death in patients with prehospital exposure to the treatment of interest. Patient counts are reported as N (%), and odd ratios with their 95% confidence intervals are presented on a logarithmic scale.

†The models were adjusted for age, sex, cardiovascular risk factors, and coexisting conditions.

In patients without prehospital exposure, the initiation of an agent acting on the renin-angiotensin system (aOR 0.14 [0.03–0.71], p = 0.018) during hospitalization was associated with a significant reduction in the odds of in-hospital death. Conversely, the initiation of a diuretic (aOR 5.19 [2.98–9.03], p<0.001) and an antiarrhythmic (aOR 6.62 [2.07–21.15], p<0.001) were associated with an increase in the odds of in-hospital death (**Table 5 and Fig 2**).

**Table 5:** Association of initiation of cardiovascular therapies with the risk of in-hospital death in patients with COVID-19 without prehospital exposure to the treatment of interest.

*Initiation (*vs. *absence)*	Initiation	Absence	Crude OR		Adjusted OR †	
	*N (%)*	*N (%)*	*(95% CI)*	*P value*	*(95% CI)*	*P value*
Agents acting on the renin-angiotensin system	2/28 (7.1)	89/542 (16.4)	0.39 (0.09–1.68)	0.207	0.14 (0.03–0.71)	0.018
ß-blocking agents	9/71 (12.7)	88/611 (14.4)	0.86 (0.41–1.80)	0.694	0.91 (0.41–2.03)	0.816
Calcium channel blockers	13/84 (15.5)	99/631 (15.7)	0.98 (0.52–1.85)	0.960	0.85 (0.42–1.72)	0.653
Diuretics	57/152 (37.5)	35/486 (7.2)	7.73 (4.81–12.44)	<0.001	5.19 (2.98–9.03)	<0.001
Lipid-modifying agents	2/21 (9.5)	97/615 (15.8)	0.56 (0.13–2.45)	0.443	0.23 (0.04–1.18)	0.078
Antiplatelets	4/27 (14.8)	100/631 (15.8)	0.92 (0.31–2.73)	0.885	0.62 (0.18–2.13)	0.447
Anticoagulants	80/606 (13.2)	15/99 (15.2)	0.85 (0.47–1.55)	0.599	0.70 (0.35–1.40)	0.309
Antiarrhythmics	9/17 (52.9)	140/805 (17.4)	5.34 (2.03–14.09)	0.001	6.62 (2.07–21.15)	0.001

Association of initiation of cardiovascular therapies with the risk of in-hospital death in patients without prehospital exposure to the treatment of interest. Patient counts are reported as N (%), and odd ratios with their 95% confidence intervals are presented on a logarithmic scale.

†The models were adjusted for age, sex, cardiovascular risk factors, and coexisting conditions.

### Laboratory values, vital signs, and in-hospital cardiovascular complications

Vital signs and laboratory values at admission for patients with modified cardiovascular therapy exposure are reported in **S1–S8 Tables**. Patients who discontinued RASi therapy had lower systolic blood pressure (SBP 119 vs. 131 mmHg, p<0.001) in contrast with those who discontinued another blood-pressure-lowering medication, such as β-blockers, calcium channel blockers, or diuretics. Conversely, patients who initiated RASis (SBP 121 vs. 137, p<0.001) and calcium channel blockers (SBP 122 vs. 126, p = 0.007) had higher SBP. Those who discontinued RASis (eGFR 73 vs. 86 mL/min/1.73m^2^, p = 0.042), β-blockers (eGFR 72 vs. 83, p = 0.026), calcium channel blockers (77 vs. 51, p<0.001), diuretics (83 vs. 74, p<0.001), and antiplatelets (eGFR 80 vs. 54, p = 0.001) and those who initiated diuretics (eGFR 68 vs. 86, p = 0.003), lipid modifying agents (66 vs. 84, p = 0.036) and antiplatelets (eGFR 65 vs. 84, p = 0.032) had significantly lower kidney function. Higher inflammation parameters were found in patients who discontinued RASis (CRP 76 vs. 53 mg/L, p = 0.002), β-blockers (WBC 9.0 vs. 5.9 G/L), diuretics (CRP 81 vs. 48, p = 0.004), and those who initiated antiarrhythmic treatment (CRP 117 vs. 53, p = 0.001; WBC 8.9 vs. 5.9, p = 0.007). The latter also had lower diastolic blood pressure (DBP 65 vs. 72 mmHg, p = 0.035).

The in-hospital occurrence of cardiovascular complications in these patients is also reported in **S1–S8 Tables**. Patients who initiated a RASi or a diuretic during hospitalization were more likely to have developed heart failure than those who did not (28.6% vs. 7.7%, p<0.001 and 19.7% vs. 2.3%, p<0.001, respectively). In addition, those who initiated an antiaggregant or a lipid-modifying agent were more likely to have developed an acute coronary syndrome (28.6% vs. 0.8%, p<0.001 and 22.2% vs. 0.3%, p<0.001, respectively). Finally, those who initiated a β-blocker or antiarrhythmic drug were more likely to have developed almost any individual cardiovascular complications.

## Discussion

In this cohort study including 838 consecutively hospitalized patients with COVID-19, we assessed the associations of exposure and early modification in exposure (i.e., discontinuation, initiation) to eight common cardiovascular therapies with the risk of in-hospital death. In a real-life setting, no benefits in terms of mortality were observed following the discontinuation of any cardiovascular drug on hospital admission. There is no evidence supporting routine discontinuation of cardiovascular medications unless clear contraindications are present. These results are consistent with previous findings in hospitalized patients with COVID-19 [17, 23, 24], and it appears sufficiently safe to advise the continuation of regular treatments acting on the cardiovascular system, as is recommended by the current guidelines [25, 26].

### Agents acting on the renin-angiotensin system

The use of RASi therapy was controversial during the early phase of the pandemic [27]. RASis were hypothesized to upregulate the expression of ACE2 receptors, whose binding with the spike protein of SARS-CoV-2 on the host cell surface was identified to trigger viral endocytosis. Current data on ACE2 upregulation following the use of RASis were issued from animal models [23]. Some observational studies, supported by a large meta-analysis of 53 studies, indicated that the use of RASis was not associated with higher in-hospital mortality or severity [18, 28, 29]. However, another meta-analysis of four randomized controlled trials did not indicate any impact of using RASis on mortality [30].

Continuation (vs. discontinuation) of RASi therapy was studied in two randomized trials: both of these studies did not indicate a significant impact of continuing this therapy on the risk of death [31, 32]. The present analysis indicated an improved survival in the group of patients who used RASis during hospitalization and did not show any benefit in RASis interruption. Furthermore, it indicated that discontinuing RASi therapy was associated with a 4-fold increase in the odds of in-hospital death. Lower blood pressure in the discontinuation group and concerns of possibly enhanced COVID-19 severity appear to be the main drivers of discontinuing these therapies. Overall, our findings support previous evidence that RASis are not associated with higher in-hospital mortality in patients with COVID-19.

Several hypotheses support the potential beneficial effects of RASis, which were found in previous observational studies. First, both ACEis and ARBs inhibit the inflammatory and prothrombotic processes associated with angiotensin II by inhibiting its binding to ACE2. Second, ACEis and ARBs promote the conversion of angiotensin I and II into angiotensin 1–9 and 1–7, which have additional anti-inflammatory and antithrombotic effects. Third, these agents have protective effects on the kidneys and heart [33]: their pleiotropic actions may prevent the development of heart failure, myocardial infarction, and acute kidney injury, which are frequent complications in patients with COVID-19 [34].

### β-blockers and calcium channel blockers

Beneficial effects of β-blockers and calcium channel blockers were hypothesized in patients with COVID-19 [35, 36]. First, β-blockers exert beneficial effects in patients without COVID-19 with sepsis, septic shock, and ARDS [37]. These conditions are also frequent and critical complications of COVID-19. Second, dihydropyridine calcium channel blockers may theoretically interfere with viral replication pathways as intracellular calcium plays an essential role in each viral replication stage [38]. Studies assessing the effect of β-blockers and calcium channel blockers on mortality in patients with COVID-19 indicated mixed effects [17, 39, 40]. We found that the discontinuation of β-blockers was associated with a 5-fold increase in the odds of in-hospital death. Neither calcium channel blockers use, nor modifications were associated with the odds of in-hospital death.

### Diuretics

Data regarding diuretics in patients with COVID-19 are scarce and mainly concern their prehospital use. Diuretics are frequently used in end-stage renal disease to treat acute kidney injuries, heart failure, or volume overload. There is no pathophysiological evidence to suggest that diuretics are a causative contributor to increased mortality, but rather an imputable consequence of COVID-19 complications with multiorgan failures requiring diuretic therapy. In our study, in-hospital use and initiation of diuretics during hospitalization, but not prehospital exposure, were associated with an increase in mortality in patients with COVID-19.

### Lipid-modifying agents

Statins, which were the most used lipid-modifying agents in this cohort, decrease oxidative stress and inflammation, promote plaque stability, improve endothelial function, and the rheological properties of erythrocytes to achieve better organ perfusion. They display immune-modulating effects and protect patients with COVID-19 from uncontrolled immune responses [41, 42]. Moreover, pausing statins could have a significant impact as a rebound phenomenon after discontinuation could facilitate inflammatory processes [43, 44].

Statins were associated with decreased mortality in observational studies, such as in a large Swedish registry-based cohort study [45], and two meta-analyses [46, 47]. However, the INSPIRATION/INSPIRATION-S randomized clinical trials investigated the initiation of atorvastatin 20 mg daily in patients admitted to intensive care. The results showed no significant benefits in terms of mortality [48]. While statins were thoroughly assessed, little is known about the effect on mortality of other lipid-modifying agents.

We found that discontinuation of lipid modifying agents resulted in a 3-fold increase in the odds of in-hospital death in patients with COVID-19. Conversely, in-hospital use of statins was associated with a significant decrease in in-hospital mortality.

### Antithrombotics

Thromboembolic events are common complications in patients with COVID-19: they were found to occur in 21% of hospitalized patients, with a 10% increase in mortality [2]. In this cohort, 27 (3.2%) patients were diagnosed with venous thromboembolism and 18 (2.1%) with acute coronary syndrome. Autopsy reports suggested that a substantial proportion of these events were not diagnosed prior to death. Drugs regulating endothelial and thrombocyte function, such as antiplatelets and anticoagulants, were deemed beneficial in patients presenting with severe COVID-19 [24].

Anticoagulation in COVID-19 has been extensively studied at various doses with converging effects, although the optimal dose and its effects for particular subgroups remain to be clarified [49]. Of interest concerning antiplatelets, the RECOVERY randomized clinical trial indicated a reduction in both 28-day mortality and the risk of progressing to invasive mechanical ventilation or death [50]. We found no association of antiplatelet use and in-hospital mortality, nor exposure modification with in-hospital mortality. Anticoagulants were associated with an increase in the odds of death if discontinued, whose main indication is bleeding.

### Strengths and limitations

We built a sequential analytical model to quantify the associations of cardiovascular therapies with mortality, accounting for detailed exposure modifications in a real-world setting. Data were prospectively collected during patient hospitalization and systematically reviewed by trained medical staff in the Department of Cardiology at the University Hospital of Geneva including symptoms and quality of life at 1-year follow-up of patient discharged after an acute COVID episode [51]. Together with specific electronic health records in COVID units, the therapy compliance was tightly monitored based on a tracking system to reduce administration errors or lack of compliance.

However, several limitations should be discussed. First, because of its observational nature, it carries the inherent limitations of any observational study, and we acknowledge that the results did not provide causal inference. Second, although key parameters such as vital signs, laboratory values, and the event of cardiovascular complications that certainly influenced clinical decisions were analyzed, the causes of treatment discontinuation and initiation could not be definitively identified, thus correction for confounding by indication was not applicable, and the results should be interpreted with caution. Third, discontinuation or initiation of cardiovascular medications occurred in patients who had already developed complications or those who have overcome the critical phase of the disease. Fourth, the consideration of individual traits (e.g., pharmacogenomics) or "wash-off" effects of single drugs, such as different medication half-lives or protracted effect of diuretics on blood pressure, could not be taken into account in the statistical analyses without strong assumptions that could further biased the interpretation. Fifth, the results of the adjusted analyses for discontinuation and initiation of a drug allowed for exposed and unexposed patients prior to hospitalization, respectively, should be interpreted with caution as they involve smaller collectives of patients and an overfitting risk is possible. Finally, these results might not apply to more recent SARS-CoV-2 variants or vaccinated patients and should thus be extrapolated with caution.

## Conclusion

This study highlights the use and modifications in prescription patterns of cardiovascular pharmacotherapy during acute COVID-19. In hospitalized patients with COVID-19, there was no detrimental association of the prehospital use of cardiovascular medication with in-hospital mortality after accounting for potential confounders. These findings support the recommendations of medical societies to continue regular cardiovascular medications, even though some drugs do not seem essential in the acute phase. Drug discontinuation might be associated with increased in-hospital mortality. While certain medications, such as agents acting on the renin-angiotensin system and lipid-modifying agents, might benefit hospitalized patients with severe COVID-19, it remains to be determined whether these medications could prevent patients from persisting COVID-19 symptoms and could improve their quality of life.

## Supporting information

S1 File Additional definitions. (PDF)

S1 Table Vital signs and laboratory values at hospital admission in patients with modified RASi exposure status with (discontinuation vs continuation) and without (absence vs initiation) prior exposure to this therapy. (PDF)

S2 Table Vital signs and laboratory values at hospital admission in patients with modified beta blocking therapy exposure status with (discontinuation vs continuation) and without (absence vs initiation) prior exposure to this therapy. (PDF)

S3 Table Vital signs and laboratory values at hospital admission in patients with modified calcium channel blocking therapy exposure status with (discontinuation vs continuation) and without (absence vs initiation) prior exposure to this therapy. (PDF)

S4 Table Vital signs and laboratory values at hospital admission in patients with modified diuretics exposure status with (discontinuation vs continuation) and without (absence vs initiation) prior exposure to this therapy. (PDF)

S5 Table Vital signs and laboratory values at hospital admission in patients with modified lipid modifying agents exposure status with (discontinuation vs continuation) and without (absence vs initiation) prior exposure to this therapy. (PDF)

S6 Table Vital signs and laboratory values at hospital admission in patients with modified antiplatelet therapy exposure status with (discontinuation vs continuation) and without (absence vs initiation) prior exposure to this therapy. (PDF)

S7 Table Vital signs and laboratory values at hospital admission in patients with modified anticoagulation exposure status with (discontinuation vs continuation) and without (absence vs initiation) prior exposure to this therapy. (PDF)

S8 Table Vital signs and laboratory values at hospital admission in patients with modified antiarrhythmics exposure status with (discontinuation vs continuation) and without (absence vs initiation) prior exposure to this therapy. (PDF)
