# Supplementary material for: Cardiovascular therapy use, modification, and in-hospital death in patients with COVID-19: A cohort study
Source: PLoS One. 2022 Nov 23;17(11):e0277653. doi: 10.1371/journal.pone.0277653 (PMC9683559; doi:10.1371/journal.pone.0277653)
Supplement: S1 File — (PDF) [file pone.0277653.s001.pdf]

Supporting information

DEFINITIONS

Cardiovascular risk factors

Cardiovascular risk factors were defined as hypertension, obesity, dyslipidemia, diabetes (type I or II), active smoking, physical inactivity, stress, and a family history of cardiovascular disease.

Coexisting conditions and history of cardiovascular disease

Coexisting conditions included cardiovascular risk factors, cardiovascular disease, respiratory disease, moderate or severe chronic kidney disease, chronic liver disease, and active cancer. Cardiovascular disease included a history of acute coronary syndrome (STEMI, NSTEMI, UA), stroke, major arrhythmias (atrial fibrillation, atrial flutter, ventricular tachycardia, or ventricular fibrillation), and heart failure. Respiratory diseases were defined as chronic obstructive pulmonary disease, asthma, and sleep apnea.

Cardiovascular complications

Cardiovascular complications were assessed as follows:

- Acute coronary syndrome: myocardial infarction (either STEMI, NSTEMI, and UA) was defined according to international guidelines [1];
- Major arrhythmia: atrial fibrillation, atrial flutter, ventricular tachycardia, ventricular fibrillation;
- Ischaemic or hemorrhagic stroke;
- Acute heart failure: transthoracic echocardiography with left ventricular ejection fraction < 50%, heart failure symptoms and elevated N-terminal-pro brain natriuretic peptide (NT-pro BNP) according to the European guidelines [2];
- Venous thromboembolic complications: pulmonary embolism and deep vein thrombosis.

COVID-19 severity

The severity of COVID-19 was classified based on an adaptation of the WHO criteria as defined in the *COVID-19 clinical management living guidance* (January 25, 2021 version) [3]:

- Mild: symptomatic patients without clinical or radiological signs of pneumonia
- Moderate: patients with clinical or radiological signs of pneumonia, without signs of severity
- Severe: patients with clinical or radiological signs of pneumonia and signs of severity (saturation < 90% on room air or respiratory rate > 30 cycles/min)
- Critical: patients with acute respiratory distress, sepsis, septic shock, acute venous thromboembolism (i.e., deep vein thrombosis, pulmonary embolism), acute coronary syndrome, or stroke.

Hospitalization criteria for patients with suspected or confirmed SARS-CoV-2 infection

The hospitalization criteria at the Geneva University Hospitals for patients with COVID-19 were determined according to the WHO guidelines (pneumonia with CURB-65 ≥ 2, oxygen dependency, tachypnoea (≥ 20/min), any organ failure, and altered conscious status). Intensive care unit admission was based on the criteria set by the Swiss Medical Society of Intensive Care Medicine. [4] However, it should be noted that these criteria were established while the pandemic was ongoing and that some patients were hospitalized without fulfilling these criteria.

REFERENCES

1. Thygesen K, Alpert JS, Jaffe AS, Chaitman BR, Bax JJ, Morrow DA, et al. Fourth Universal Definition of Myocardial Infarction (2018). Journal of the American College of Cardiology. 2018;72(18):2231-64.

2. Ponikowski P, Voors AA, Anker SD, Bueno H, Cleland JGF, Coats AJS, et al. 2016 ESC Guidelines for the diagnosis and treatment of acute and chronic heart failure: The Task Force for the diagnosis and treatment of acute and chronic heart failure of the European Society of Cardiology (ESC)Developed with the special contribution of the Heart Failure Association (HFA) of the ESC. European Heart Journal. 2016;37(27):2129-200.

3. World Health O. COVID-19 clinical management: living guidance, 25 January 2021. Geneva: World Health Organization; 2021 2021. Contract No.: WHO/2019-nCoV/clinical/2021.1.

4. Swiss Academy Of Medical S. COVID-19 pandemic: triage for intensive-care treatment under resource scarcity. Swiss Med Wkly. 2020;150:w20229.
