# Supplementary material for: Cardiovascular therapy use, modification, and in-hospital death in patients with COVID-19: A cohort study
Source: PLoS One. 2022 Nov 23;17(11):e0277653. doi: 10.1371/journal.pone.0277653 (PMC9683559; doi:10.1371/journal.pone.0277653)
Supplement: S1 Table — (PDF) [file pone.0277653.s002.pdf]

Supporting information

S1 Table. Vital signs and laboratory values at hospital admission in patients with modified RASi exposure status with (discontinuation vs continuation) and without (absence vs initiation) prior exposure to this therapy.

	**Continuation vs discontinuation**				**Initiation vs absence**			
**RASi**	**Continuation**	**Discontinuation**	P value	Missings	**Absent**	**Initiation**	P value	Missings
N (%)	182 (67.9)	86 (32.1)			542 (95.1)	28 (4.9)		
Vital signs on admission								
SBP (mmHg)	131 (31)	119 (31)	<0.001	12 (4.48)	121 (27)	137 (28)	<0.001	26 (4.56)
DBP (mmHg)	72 (21)	68 (20)	0.022	12 (4.48)	72 (16)	73 (28)	0.712	26 (4.56)
Pulse (bpm)	76 (24)	78 (19)	0.249	12 (4.48)	78 (25)	70 (36)	0.356	26 (4.56)
Respiratory rate (cpm)	22 (7)	23 (8)	0.148	16 (5.97)	21 (7)	21 (6)	0.521	42 (7.37)
Laboratory on admission								
WBC (G/L)	6.1 (3.6)	6.2 (4.0)	0.745	5 (1.87)	5.7 (3.5)	6.5 (4.0)	0.108	19 (3.33)
CRP (mg/L)	53.1 (76.1)	76.2 (64.7)	0.002	10 (3.73)	51.6 (74.3)	66.6 (124.6)	0.323	24 (4.21)
eGFR (CKD-EPI) (mL/min/1.73m2)	61.9 (39.5)	52.9 (37.2)	0.003	4 (1.49)	86.0 (34.6)	72.7 (52.4)	0.042	17 (2.98)
Creatinin (µmol/L),	93.0 (46.5)	108.0 (80.0)	0.002	4 (1.49)	75.5 (30.0)	87.0 (41.0)	0.068	17 (2.98)
Outcomes								
Cardiovascular events (overall)	52 (28.6)	18 (20.9)	0.184	0 (0.00)	77 (14.2)	13 (46.4)	<0.001	0 (0.00)
Acute coronary syndrome	7 (3.8)	2 (2.3)	0.519	0 (0.00)	7 (1.3)	2 (7.1)	0.015	0 (0.00)
Arrhythmia	16 (8.8)	5 (5.8)	0.397	0 (0.00)	21 (3.9)	3 (10.7)	0.079	0 (0.00)
Heart failure	28 (15.4)	11 (12.8)	0.574	0 (0.00)	42 (7.7)	8 (28.6)	<0.001	0 (0.00)
Stroke	5 (2.7)	1 (1.2)	0.413	0 (0.00)	4 (0.7)	0 (0.0)	0.648	0 (0.00)
Acute venous thromboembolism	4 (2.2)	4 (4.7)	0.271	0 (0.00)	17 (3.1)	2 (7.1)	0.249	0 (0.00)

Data are expressed as median with interquartile range for continuous variables and count with relative percentage for missing values. P-values were obtained using the Wilcoxon-Mann-Whitney test. SBP: systolic blood pressure; DBP: diastolic blood pressure; WBC: white blood cells; CRP: C reactive protein; eGFR estimated glomerular filtration rate.
