# Supplementary material for: Cardiovascular therapy use, modification, and in-hospital death in patients with COVID-19: A cohort study
Source: PLoS One. 2022 Nov 23;17(11):e0277653. doi: 10.1371/journal.pone.0277653 (PMC9683559; doi:10.1371/journal.pone.0277653)
Supplement: S2 Table — (PDF) [file pone.0277653.s003.pdf]

Supporting information

S2 Table. Vital signs and laboratory values at hospital admission in patients with modified beta blocking therapy exposure status with (discontinuation vs continuation) and without (absence vs initiation) prior exposure to this therapy.

	**Continuation vs discontinuation**				**Initiation vs absence**			
**Beta-blocking agents**	**Continuation**	**Discontinuation**	P value	Missings	**Absent**	**Initiation**	P value	Missings
N (%)	142 (91.0)	14 (9.0)			611 (89.6)	71 (10.4)		
Vital signs on admission								
SBP (mmHg)	125 (29)	122 (43)	0.319	11 (7.05)	123 (28)	125 (30)	0.488	27 (3.96)
DBP (mmHg)	70 (20)	70 (28)	0.802	11 (7.05)	72 (16)	72 (22)	0.477	27 (3.96)
Pulse (bpm)	78 (25)	64 (82)	0.064	11 (7.05)	78 (24)	79 (27)	0.503	27 (3.96)
Respiratory rate (cpm)	21 (8)	24 (8)	0.383	15 (9.62)	22 (8)	22 (8)	0.830	43 (6.30)
Laboratory on admission								
WBC (G/L)	5.9 (4.3)	9.0 (3.7)	0.023	7 (4.49)	5.8 (3.3)	6.7 (4.6)	0.004	17 (2.49)
CRP (mg/L)	49.5 (62.8)	68.8 (100.7)	0.269	7 (4.49)	54.1 (79.6)	79.5 (103.8)	0.040	27 (3.96)
eGFR (mL/min/1.73m2)	51.4 (49.0)	65.7 (43.9)	0.393	4 (2.56)	82.8 (36.9)	72.3 (33.1)	0.026	17 (2.49)
Creatinin (µmol/L),	99.5 (94.5)	89.5 (67.5)	0.431	4 (2.56)	77.0 (34.2)	81.0 (38.0)	0.008	17 (2.49)
Outcomes								
Cardiovascular events (overall)	60 (42.3)	5 (35.7)	0.636	0 (0.00)	60 (9.8)	35 (49.3)	<0.001	0 (0.00)
Acute coronary syndrome	7 (4.9)	0 (0.0)	0.395	0 (0.00)	7 (1.1)	4 (5.6)	0.004	0 (0.00)
Arrhythmia	16 (11.3)	1 (7.1)	0.637	0 (0.00)	11 (1.8)	17 (23.9)	<0.001	0 (0.00)
Heart failure	39 (27.5)	5 (35.7)	0.513	0 (0.00)	31 (5.1)	14 (19.7)	<0.001	0 (0.00)
Stroke	3 (2.1)	0 (0.0)	0.583	0 (0.00)	3 (0.5)	4 (5.6)	<0.001	0 (0.00)
Acute venous thromboembolism	6 (4.2)	0 (0.0)	0.433	0 (0.00)	14 (2.3)	7 (9.9)	<0.001	0 (0.00)

Data are expressed as median with interquartile range for continuous variables and count with relative percentage for missing values. P-values were obtained using the Wilcoxon-Mann-Whitney test. SBP: systolic blood pressure; DBP: diastolic blood pressure; WBC: white blood cells; CRP: C reactive protein; eGFR estimated glomerular filtration rate.
