# Supplementary material for: Cardiovascular therapy use, modification, and in-hospital death in patients with COVID-19: A cohort study
Source: PLoS One. 2022 Nov 23;17(11):e0277653. doi: 10.1371/journal.pone.0277653 (PMC9683559; doi:10.1371/journal.pone.0277653)
Supplement: S3 Table — (PDF) [file pone.0277653.s004.pdf]

Supporting information

S3 Table. Vital signs and laboratory values at hospital admission in patients with modified calcium channel blocking therapy exposure status with (discontinuation vs continuation) and without (absence vs initiation) prior exposure to this therapy.

	**Continuation vs discontinuation**				**Initiation vs absence**			
**Calcium channel blockers**	**Continuation**	**Discontinuation**	P value	Missings	**Absent**	**Initiation**	P value	Missings
N (%)	93 (75.6)	30 (24.4)			631 (88.3)	84 (11.7)		
Vital signs on admission								
SBP (mmHg)	133 (38)	127 (45)	0.170	7 (5.69)	122 (26)	126 (31)	0.004	31 (4.34)
DBP (mmHg)	70 (25)	70 (20)	0.747	7 (5.69)	72 (16)	74 (21)	0.361	31 (4.34)
Pulse (bpm)	78 (26)	76 (21)	0.633	7 (5.69)	78 (24)	78 (28)	0.818	31 (4.34)
Respiratory rate (cpm)	22 (9)	22 (8)	0.681	10 (8.13)	22 (8)	21 (8)	0.440	48 (6.71)
Laboratory on admission								
WBC (G/L)	6.3 (4.1)	6.7 (4.5)	0.871	4 (3.25)	5.8 (3.6)	6.2 (3.5)	0.113	20 (2.80)
CRP (mg/L)	54.5 (71.8)	55.1 (106.6)	0.665	4 (3.25)	50.5 (74.3)	76.5 (96.6)	0.001	30 (4.20)
eGFR (mL/min/1.73m2)	58.1 (49.7)	47.4 (41.4)	0.055	2 (1.63)	82.5 (38.7)	76.7 (32.1)	0.058	19 (2.66)
Creatinin (µmol/L),	97.0 (79.0)	109.5 (123.8)	0.071	2 (1.63)	77.0 (36.0)	79.0 (40.5)	0.294	19 (2.66)
Outcomes								
Cardiovascular events (overall)	25 (26.9)	10 (33.3)	0.496	0 (0.00)	105 (16.6)	20 (23.8)	0.104	0 (0.00)
Acute coronary syndrome	2 (2.2)	0 (0.0)	0.418	0 (0.00)	13 (2.1)	3 (3.6)	0.379	0 (0.00)
Arrhythmia	5 (5.4)	3 (10.0)	0.372	0 (0.00)	31 (4.9)	6 (7.1)	0.386	0 (0.00)
Heart failure	11 (11.8)	7 (23.3)	0.121	0 (0.00)	63 (10.0)	8 (9.5)	0.895	0 (0.00)
Stroke	2 (2.2)	1 (3.3)	0.715	0 (0.00)	4 (0.6)	3 (3.6)	0.010	0 (0.00)
Acute venous thromboembolism	5 (5.4)	1 (3.3)	0.651	0 (0.00)	14 (2.2)	7 (8.3)	0.002	0 (0.00)

Data are expressed as median with interquartile range for continuous variables and count with relative percentage for missing values. P-values were obtained using the Wilcoxon-Mann-Whitney test. SBP: systolic blood pressure; DBP: diastolic blood pressure; WBC: white blood cells; CRP: C reactive protein; eGFR estimated glomerular filtration rate.
