# Supplementary material for: Cardiovascular therapy use, modification, and in-hospital death in patients with COVID-19: A cohort study
Source: PLoS One. 2022 Nov 23;17(11):e0277653. doi: 10.1371/journal.pone.0277653 (PMC9683559; doi:10.1371/journal.pone.0277653)
Supplement: S4 Table — (PDF) [file pone.0277653.s005.pdf]

Supporting information

S4 Table. Vital signs and laboratory values at hospital admission in patients with modified diuretics exposure status with (discontinuation vs continuation) and without (absence vs initiation) prior exposure to this therapy.

	**Continuation vs discontinuation**				**Initiation vs absence**			
**Diuretics**	**Continuation**	**Discontinuation**	P value	Missings	**Absent**	**Initiation**	P value	Missings
N (%)	153 (76.5)	47 (23.5)			486 (76.2)	152 (23.8)		
Vital signs on admission								
SBP (mmHg)	126 (34)	119 (32)	0.120	11 (5.50)	123 (27)	124 (28)	0.778	27 (4.23)
DBP (mmHg)	68 (23)	70 (18)	0.419	11 (5.50)	74 (17)	70 (16)	0.001	27 (4.23)
Pulse (bpm)	75 (25)	78 (19)	0.099	11 (5.50)	78 (23)	79 (26)	0.839	27 (4.23)
Respiratory rate (cpm)	22 (9)	21 (6)	0.251	18 (9.00)	21 (7)	22 (8)	0.106	40 (6.27)
Laboratory on admission								
WBC (G/L)	6.1 (4.4)	6.6 (3.8)	0.211	6 (3.00)	5.6 (3.2)	6.7 (4.2)	<0.001	18 (2.82)
CRP (mg/L)	48.0 (63.5)	81.3 (94.5)	0.024	7 (3.50)	48.2 (71.9)	81.7 (102.6)	<0.001	27 (4.23)
eGFR (mL/min/1.73m2)	47.8 (42.0)	50.4 (31.3)	0.597	4 (2.00)	88.2 (31.1)	69.8 (35.5)	<0.001	17 (2.66)
Creatinin (µmol/L),	105.0 (81.0)	111.0 (67.5)	0.376	4 (2.00)	74.0 (30.0)	83.0 (40.0)	<0.001	17 (2.66)
Outcomes								
Cardiovascular events (overall)	64 (41.8)	10 (21.3)	0.011	0 (0.00)	32 (6.6)	54 (35.5)	<0.001	0 (0.00)
Acute coronary syndrome	6 (3.9)	1 (2.1)	0.558	0 (0.00)	4 (0.8)	7 (4.6)	0.002	0 (0.00)
Arrhythmia	13 (8.5)	3 (6.4)	0.640	0 (0.00)	13 (2.7)	16 (10.5)	<0.001	0 (0.00)
Heart failure	44 (28.8)	4 (8.5)	0.004	0 (0.00)	11 (2.3)	30 (19.7)	<0.001	0 (0.00)
Stroke	2 (1.3)	2 (4.3)	0.207	0 (0.00)	4 (0.8)	2 (1.3)	0.583	0 (0.00)
Acute venous thromboembolism	7 (4.6)	1 (2.1)	0.454	0 (0.00)	5 (1.0)	14 (9.2)	<0.001	0 (0.00)

Data are expressed as median with interquartile range for continuous variables and count with relative percentage for missing values. P-values were obtained using the Wilcoxon-Mann-Whitney test. SBP: systolic blood pressure; DBP: diastolic blood pressure; WBC: white blood cells; CRP: C reactive protein; eGFR estimated glomerular filtration rate.
