# Supplementary material for: Cardiovascular therapy use, modification, and in-hospital death in patients with COVID-19: A cohort study
Source: PLoS One. 2022 Nov 23;17(11):e0277653. doi: 10.1371/journal.pone.0277653 (PMC9683559; doi:10.1371/journal.pone.0277653)
Supplement: S5 Table — (PDF) [file pone.0277653.s006.pdf]

Supporting information

S5 Table. Vital signs and laboratory values at hospital admission in patients with modified lipid modifying agents exposure status with (discontinuation vs continuation) and without (absence vs initiation) prior exposure to this therapy.

	**Continuation vs discontinuation**				**Initiation vs absence**			
**Lipid modifying agents**	**Continuation**	**Discontinuation**	P value	Missings	**Absent**	**Initiation**	P value	Missings
N (%)	148 (73.3)	54 (26.7)			615 (96.7)	21 (3.3)		
Vital signs on admission								
SBP (mmHg)	126 (35)	123 (40)	0.429	8 (3.96)	123 (26)	124 (44)	0.671	30 (4.72)
DBP (mmHg)	67 (18)	70 (20)	0.580	8 (3.96)	73 (16)	68 (20)	0.078	30 (4.72)
Pulse (bpm)	76 (23)	77 (30)	0.976	8 (3.96)	78 (24)	70 (46)	0.012	30 (4.72)
Respiratory rate (cpm)	21 (8)	23 (7)	0.048	10 (4.95)	22 (8)	20 (5)	0.581	48 (7.55)
Laboratory on admission								
WBC (G/L)	6.2 (4.3)	6.1 (4.4)	0.786	4 (1.98)	5.8 (3.6)	7.1 (4.3)	0.083	20 (3.14)
CRP (mg/L)	49.5 (68.9)	58.8 (104.8)	0.296	6 (2.97)	55.0 (77.2)	54.6 (51.5)	0.799	28 (4.40)
eGFR (mL/min/1.73m2)	54.5 (44.1)	61.1 (50.0)	0.331	2 (0.99)	83.8 (36.4)	66.1 (40.5)	0.036	19 (2.99)
Creatinin (µmol/L),	97.0 (75.0)	94.0 (82.5)	0.367	2 (0.99)	76.0 (34.0)	93.0 (43.5)	0.006	19 (2.99)
Outcomes								
Cardiovascular events (overall)	48 (32.4)	11 (20.4)	0.095	0 (0.00)	89 (14.5)	12 (57.1)	<0.001	0 (0.00)
Acute coronary syndrome	5 (3.4)	2 (3.7)	0.911	0 (0.00)	5 (0.8)	6 (28.6)	<0.001	0 (0.00)
Arrhythmia	14 (9.5)	2 (3.7)	0.180	0 (0.00)	25 (4.1)	4 (19.0)	0.001	0 (0.00)
Heart failure	29 (19.6)	9 (16.7)	0.637	0 (0.00)	47 (7.6)	4 (19.0)	0.058	0 (0.00)
Stroke	6 (4.1)	1 (1.9)	0.449	0 (0.00)	3 (0.5)	0 (0.0)	0.748	0 (0.00)
Acute venous thromboembolism	2 (1.4)	0 (0.0)	0.391	0 (0.00)	23 (3.7)	2 (9.5)	0.180	0 (0.00)

Data are expressed as median with interquartile range for continuous variables and count with relative percentage for missing values. P-values were obtained using the Wilcoxon-Mann-Whitney test. SBP: systolic blood pressure; DBP: diastolic blood pressure; WBC: white blood cells; CRP: C reactive protein; eGFR estimated glomerular filtration rate.
