# Supplementary material for: Cardiovascular therapy use, modification, and in-hospital death in patients with COVID-19: A cohort study
Source: PLoS One. 2022 Nov 23;17(11):e0277653. doi: 10.1371/journal.pone.0277653 (PMC9683559; doi:10.1371/journal.pone.0277653)
Supplement: S6 Table — (PDF) [file pone.0277653.s007.pdf]

Supporting information

S6 Table. Vital signs and laboratory values at hospital admission in patients with modified antiplatelet therapy exposure status with (discontinuation vs continuation) and without (absence vs initiation) prior exposure to this therapy.

	**Continuation vs discontinuation**				**Initiation vs absence**			
**Antiplatelets**	**Continuation**	**Discontinuation**	P value	Missings	**Absent**	**Initiation**	P value	Missings
N (%)	154 (85.6)	26 (14.4)			631 (95.9)	27 (4.1)		
Vital signs on admission								
SBP (mmHg)	128 (36)	136 (44)	0.234	11 (6.11)	122 (26)	125 (52)	0.797	27 (4.10)
DBP (mmHg)	69 (17)	72 (24)	0.181	11 (6.11)	73 (16)	72 (26)	0.524	27 (4.10)
Pulse (bpm)	76 (24)	66 (80)	0.135	11 (6.11)	78 (24)	73 (88)	0.206	27 (4.10)
Respiratory rate (cpm)	21 (6)	22 (10)	0.526	14 (7.78)	22 (8)	23 (7)	0.236	44 (6.69)
Laboratory on admission								
WBC (G/L)	5.8 (4.3)	7.0 (4.0)	0.281	6 (3.33)	5.8 (3.5)	6.9 (3.8)	0.229	18 (2.74)
CRP (mg/L)	40.6 (71.6)	50.9 (63.1)	0.411	6 (3.33)	56.6 (75.2)	78.0 (101.6)	0.053	28 (4.26)
eGFR (mL/min/1.73m2)	53.8 (43.0)	80.3 (34.1)	0.001	4 (2.22)	83.5 (36.9)	64.9 (53.0)	0.032	17 (2.58)
Creatinin (µmol/L),	103.0 (78.8)	72.0 (34.0)	0.002	4 (2.22)	77.0 (35.0)	91.0 (52.2)	0.024	17 (2.58)
Outcomes								
Cardiovascular events (overall)	44 (28.6)	9 (34.6)	0.532	0 (0.00)	95 (15.1)	12 (44.4)	<0.001	0 (0.00)
Acute coronary syndrome	8 (5.2)	2 (7.7)	0.607	0 (0.00)	2 (0.3)	6 (22.2)	<0.001	0 (0.00)
Arrhythmia	9 (5.8)	2 (7.7)	0.716	0 (0.00)	31 (4.9)	3 (11.1)	0.154	0 (0.00)
Heart failure	26 (16.9)	6 (23.1)	0.445	0 (0.00)	52 (8.2)	5 (18.5)	0.063	0 (0.00)
Stroke	4 (2.6)	1 (3.8)	0.720	0 (0.00)	4 (0.6)	1 (3.7)	0.072	0 (0.00)
Acute venous thromboembolism	4 (2.6)	2 (7.7)	0.181	0 (0.00)	20 (3.2)	1 (3.7)	0.877	0 (0.00)

Data are expressed as median with interquartile range for continuous variables and count with relative percentage for missing values. P-values were obtained using the Wilcoxon-Mann-Whitney test. SBP: systolic blood pressure; DBP: diastolic blood pressure; WBC: white blood cells; CRP: C reactive protein; eGFR estimated glomerular filtration rate.
