# Supplementary material for: Cardiovascular therapy use, modification, and in-hospital death in patients with COVID-19: A cohort study
Source: PLoS One. 2022 Nov 23;17(11):e0277653. doi: 10.1371/journal.pone.0277653 (PMC9683559; doi:10.1371/journal.pone.0277653)
Supplement: S7 Table — (PDF) [file pone.0277653.s008.pdf]

Supporting information

S7 Table. Vital signs and laboratory values at hospital admission in patients with modified anticoagulation exposure status with (discontinuation vs continuation) and without (absence vs initiation) prior exposure to this therapy.

	**Continuation vs discontinuation**				**Initiation vs absence**			
**Anticoagulants**	**Continuation**	**Discontinuation**	P value	Missings	**Absent**	**Initiation**	P value	Missings
N (%)	120 (90.2)	13 (9.8)			99 (14.0)	606 (86.0)		
Vital signs on admission								
SBP (mmHg)	130 (33)	137 (42)	0.444	7 (5.26)	120 (27)	123 (28)	0.229	31 (4.40)
DBP (mmHg)	68 (20)	63 (24)	0.857	7 (5.26)	73 (18)	72 (16)	0.877	31 (4.40)
Pulse (bpm)	78 (28)	88 (10)	0.031	7 (5.26)	74 (28)	78 (23)	0.137	31 (4.40)
Respiratory rate (cpm)	21 (8)	22 (12)	0.685	14 (10.53)	22 (6)	22 (8)	0.933	44 (6.24)
Laboratory on admission								
WBC (G/L)	5.9 (4.9)	7.0 (5.2)	0.761	5 (3.76)	6.0 (4.7)	5.8 (3.4)	1.000	19 (2.70)
CRP (mg/L)	49.8 (77.9)	56.6 (81.0)	0.803	11 (8.27)	49.8 (70.3)	55.1 (78.0)	0.180	23 (3.26)
eGFR (mL/min/1.73m2)	63.2 (46.5)	58.2 (40.1)	0.425	4 (3.01)	83.7 (39.1)	80.3 (39.0)	0.255	17 (2.41)
Creatinin (µmol/L),	92.5 (70.8)	101.0 (103.5)	0.436	4 (3.01)	75.5 (35.2)	79.5 (36.2)	0.293	17 (2.41)
Outcomes								
Cardiovascular events (overall)	47 (39.2)	7 (53.8)	0.306	0 (0.00)	8 (8.1)	98 (16.2)	0.037	0 (0.00)
Acute coronary syndrome	3 (2.5)	1 (7.7)	0.298	0 (0.00)	3 (3.0)	11 (1.8)	0.422	0 (0.00)
Arrhythmia	7 (5.8)	3 (23.1)	0.025	0 (0.00)	1 (1.0)	34 (5.6)	0.051	0 (0.00)
Heart failure	33 (27.5)	4 (30.8)	0.803	0 (0.00)	6 (6.1)	46 (7.6)	0.589	0 (0.00)
Stroke	5 (4.2)	0 (0.0)	0.453	0 (0.00)	0 (0.0)	5 (0.8)	0.364	0 (0.00)
Acute venous thromboembolism	4 (3.3)	0 (0.0)	0.504	0 (0.00)	1 (1.0)	22 (3.6)	0.174	0 (0.00)

Data are expressed as median with interquartile range for continuous variables and count with relative percentage for missing values. P-values were obtained using the Wilcoxon-Mann-Whitney test. SBP: systolic blood pressure; DBP: diastolic blood pressure; WBC: white blood cells; CRP: C reactive protein; eGFR estimated glomerular filtration rate.
