# Supplementary material for: Cardiovascular therapy use, modification, and in-hospital death in patients with COVID-19: A cohort study
Source: PLoS One. 2022 Nov 23;17(11):e0277653. doi: 10.1371/journal.pone.0277653 (PMC9683559; doi:10.1371/journal.pone.0277653)
Supplement: S8 Table — (PDF) [file pone.0277653.s009.pdf]

Supporting information

S8 Table. Vital signs and laboratory values at hospital admission in patients with modified antiarrhythmics exposure status with (discontinuation vs continuation) and without (absence vs initiation) prior exposure to this therapy.

	**Continuation vs discontinuation**				**Initiation vs absence**			
**Antiarrhythmics**	**Continuation**	**Discontinuation**	P value	Missings	**Absent**	**Initiation**	P value	Missings
N (%)	13 (81.2)	3 (18.8)			805 (97.9)	17 (2.1)		
Vital signs on admission								
SBP (mmHg)	133 (48)	134 (65)	0.427	1 (6.25)	124 (28)	122 (28)	0.315	37 (4.50)
DBP (mmHg)	58 (22)	83 (21)	0.083	1 (6.25)	72 (17)	65 (17)	0.035	37 (4.50)
Pulse (bpm)	70 (16)	79 (20)	0.420	1 (6.25)	78 (25)	76 (23)	0.301	37 (4.50)
Respiratory rate (cpm)	19 (6)	20 (5)	0.665	1 (6.25)	22 (8)	22 (8)	0.579	57 (6.93)
Laboratory on admission								
WBC (G/L)	4.9 (4.6)	7.1 (4.8)	0.243	2 (12.50)	5.9 (3.5)	8.9 (5.2)	0.007	22 (2.68)
CRP (mg/L)	48.5 (58.9)	87.0 (44.5)	0.076	3 (18.75)	53.0 (75.2)	117.1 (126.2)	0.001	31 (3.77)
eGFR (mL/min/1.73m2)	44.6 (39.9)	71.9 (60.9)	0.122	0 (0.00)	79.0 (39.7)	70.7 (29.3)	0.256	21 (2.55)
Creatinin (µmol/L),	128.0 (80.0)	91.0 (45.0)	0.093	0 (0.00)	79.0 (40.0)	97.0 (32.5)	0.208	21 (2.55)
Outcomes								
Cardiovascular events (overall)	5 (38.5)	1 (33.3)	0.869	0 (0.00)	141 (17.5)	13 (76.5)	<0.001	0 (0.00)
Acute coronary syndrome	1 (7.7)	0 (0.0)	0.620	0 (0.00)	15 (1.9)	2 (11.8)	0.005	0 (0.00)
Arrhythmia	2 (15.4)	0 (0.0)	0.468	0 (0.00)	35 (4.3)	8 (47.1)	<0.001	0 (0.00)
Heart failure	3 (23.1)	1 (33.3)	0.712	0 (0.00)	80 (9.9)	5 (29.4)	0.009	0 (0.00)
Stroke	1 (7.7)	0 (0.0)	0.620	0 (0.00)	8 (1.0)	1 (5.9)	0.055	0 (0.00)
Acute venous thromboembolism	0 (0.0)	0 (0.0)	.	0 (0.00)	23 (2.9)	4 (23.5)	<0.001	0 (0.00)

Data are expressed as median with interquartile range for continuous variables and count with relative percentage for missing values. P-values were obtained using the Wilcoxon-Mann-Whitney test. SBP: systolic blood pressure; DBP: diastolic blood pressure; WBC: white blood cells; CRP: C reactive protein; eGFR estimated glomerular filtration rate.
